# Acute Cardiorespiratory Responses to Different Exercise Modalities in Chronic Heart Failure Patients—A Pilot Study

**DOI:** 10.3390/jcdd8120164

**Published:** 2021-11-26

**Authors:** Eleftherios Karatzanos, Panagiotis Ferentinos, Georgios Mitsiou, Stavros Dimopoulos, Argyrios Ntalianis, Serafeim Nanas

**Affiliations:** 1 Clinical Ergospirometry, Exercise and Rehabilitation Laboratory, School of Medicine, National and Kapodistrian University of Athens, 106 75 Athens, Greece; gdmitsiou@gmail.com (G.M.); stdimop@gmail.com (S.D.); sernanas@gmail.com (S.N.); 2 Carnegie Faculty, School of Sport, Leeds Beckett University, Leeds LS6 3QT, UK; P.Ferentinos@leedsbeckett.ac.uk; 3 Heart Failure and Cardio-Oncology Unit, Alexandra Hospital, 115 28 Athens, Greece; arg_nt@yahoo.gr

**Keywords:** cardiac rehabilitation, heart failure, continuous exercise, high intensity interval training, acute exercise

## Abstract

The purpose of this study was to compare the acute cardiorespiratory responses and time spent above different %VO_2peak_ intensities between three “iso-work” protocols: (a) a high intensity interval training protocol (HIIT), (b) a higher intensity continuous protocol (CON_70_) and (c) a lower intensity continuous protocol (CON_50_) in patients with chronic heart failure (CHF). Ten male CHF patients (aged 55.1 ± 16.2 years) performed in separate days a single session of a HIIT protocol consisted of 4 sets × 4 min cycling at 80% VO_2peak_ with 3 min of recovery at 50% VO_2peak_, a CON_70_ protocol corresponding to 70% VO_2peak_ and a CON_50_ protocol corresponding to 50% VO_2peak_. Cardiopulmonary data were collected by an online gas analysis system. The HIIT and CON_70_ elicited higher cardiorespiratory responses compared to CON_50_ with no differences between them (*p* > 0.05). In HIIT and CON_70_, patients exercised longer at >80% and >90% VO_2peak_. The completion rate was 100% for the three protocols. Not any adverse events were observed in either protocol. Both HIIT and CON_70_ elicited a stronger physiological stimulus and required shorter time than CON_50_. Both HIIT and CON_70_ also induced comparable hemodynamic responses and ventilatory demand.

## 1. Introduction

Chronic heart failure (CHF) is a complex clinical syndrome with a prevalence of more than 23 million people globally [1]. Aerobic exercise training is an important non–pharmacological tool, which leads to ventilatory, cardiovascular, skeletal muscle and neurohormonal adaptations that benefit CHF patients [2]. These adaptations lead to an improvement in exercise capacity and health-related quality of life. The latest guidelines from the European Society of Cardiology recommend aerobic exercise as an evidence-based intervention for the management of CHF patients [3].

Exercise intensity is a fundamental component of aerobic exercise training. Peak oxygen uptake (VO_2peak_) is considered the gold standard of cardiorespiratory fitness and a predictor of cardiac related mortality and hospitalization [4]. It has been suggested that in order to increase VO_2peak_, exercise intensity is an important determinant in cardiac patients [5]. In order to achieve that, high intensity interval training (HIIT) has gained popularity in cardiac rehabilitation in recent years. A potential benefit from HIIT, shown to be similarly safe as moderate intensity continuous training (MCT) [6,7], is that more time is spent at high intensities and this greater stimulus may lead to better cardiovascular adaptations [8].

Several single centre randomized studies have observed that HIIT elicited better improvements in aerobic exercise capacity, quality of life and pathophysiological mechanisms compared to moderate continuous training [9,10,11,12]. On the other hand, two large multicenter studies (SMARTEX-HF and SAINTEX-CAD) revealed similar improvements between HIIT and MCT in CHF and coronary artery disease patients, respectively [13,14]. In both studies, however, MCT patients exercised at intensities rather higher than moderate. Furthermore, there is a wide range in the prescribed intensity of the MCT protocols in CHF studies [10,12,15,16], which make it even more difficult to suggest which exercise modality is best for CHF patients. In addition, a recent meta-analysis showed that HIIT elicited larger improvements in VO_2peak_ compared to MCT in patients with CHF but in sub-group analysis with iso-caloric exercise protocols, HIIT did not differ to MCT [11].

Previous studies have also examined the acute effects of various HIIT protocols in cardiac patients [17,18,19]. When comparing the acute effects of HIIT with an iso-caloric MCT protocol in cardiac patients, no differences have been identified based on the rate of perceived exertion, markers of cardiac damage, cardiorespiratory and hemodynamic responses [20,21]. However, all these studies utilized HIIT protocols with intervals lasting from 10 to 90 s interspersed by segments of passive recovery. Recently, a study [22] examined the acute effects between a time matched short-stage HIIT (20 s), a long-stage HIIT (4 min) and an MCT in coronary artery disease patients. Long interval HIIT induced higher peak lactate, peak VO_2_ compared to other two protocols with no significant differences being found on catecholamines, cardiac and inflammatory markers. To our knowledge, no study has examined the acute effects of a long-stage and high volume HIIT protocol in CHF patients. Moreover, all the studies compared a HIIT protocol with a single MCT. The acute effects of different intensity continuous protocols are not known yet, which could possibly provide some explanation why some studies did not find differences between HIIT and continuous exercise.

The aim of this pilot study was to compare a long duration HIIT, widely employed in recent years, with two aerobic continuous training protocols differing in intensity. It was hypothesized that a higher intensity continuous protocol would result in higher cardiorespiratory responses than a lower intensity continuous one, while it would be able to induce similar responses compared to HIIT protocol.

## 2. Materials and Methods

### 2.1. Population and STUDY Design

The pilot study recruited 10 stable consecutive CHF male patients at optimal medical treatment and LVEF less than 45% (Table 1). Exclusion criteria from the study were unstable CHF, chronic obstructive pulmonary disease, unstable angina, intermittent claudication and orthopedic conditions. The patients were enrolled after referral from their cardiologists to our Cardiopulmonary Exercise Testing and Rehabilitation Centre for participation in a cardiac rehabilitation program. Written informed consent was obtained from every participant. Ethical approval for the study was obtained from the bioethics committee of the Evaggelismos General Hospital of Athens and the study design complied with the ethical principles of the Helsinki Declaration.

The study design involved a cardiopulmonary exercise test (CPX) followed by three exercise training sessions; these were a HIIT regime, a low intensity continuous regime (CON_50_) and a higher intensity continuous regime (CON_70_). Exercise training protocols were performed in random order, with 3–4 (days) separating each-other.

### 2.2. Cardiopulmonary Exercise Test

Patients in a fasted state performed a symptom limited (CPX) to assess their baseline functional capacity and prescribe the exercise intensities of the protocols. CPX details have been previously provided [23]. Resting electrocardiogram (ECG) and heart rate were recorded, and blood pressure was measured in seated position. In short, the patients underwent an incremental ramp exercise test on an electromagnetically braked cycle ergometer (Ergoline 800, Sensor Medics, Anaheim, CA, USA)) until exhaustion using an online gas analysis system (Quark CPET, Cosmed, Rome, Italy). The work rates were calculated individually using the Hansen et al. equation [24]. VO_2peak_ was calculated as the highest 20 s average of the data [25]. Anaerobic threshold (AT) was determined using the V slope method [26] and graphs were created of ventilatory equivalent for oxygen (VE/VO_2_), carbon dioxide (VE/VCO_2_), end tidal O_2_ (PetO_2_) and CO_2_ tension (PetCO_2_) against workload. Regression analyses (workload-VO_2_) were performed to prescribe workload for the HIIT, CON_70_ and CON_50_ protocol [27].

### 2.3. Exercise Training Protocols

The HIIT protocol utilized in this study was adopted and modified from a previous study [9]. Power output was preferred over heart rate to control for intensity; the use of heart rate as a surrogate measure of workload has been suggested as unreliable in HF patients because of the high prevalence of chronic atrial fibrillation and frequent chronotropic incompetence due to maximally titrated beta blockers these patients receive [28]. Therefore, the workload (W) that corresponds with a percentage of VO_2peak_ was employed to prescribe intensity. In the HIIT protocol, after 7 min of warm up at 45% VO_2peak,_ 3 min at 50% VO_2peak_ followed by 4 sets of 4 min at 80% VO_2peak_ with 3 min active recovery at 50% VO_2peak_. In CON_70_ and CON_50_, patients exercised at 70% and 50% VO_2peak_, respectively. The total workload was calculated to be similar in all three protocols. All protocols also included a 7 min warm up at 45% VO_2peak_.

### 2.4. Data Collection

In all exercise protocols the patients were connected to a 12 lead ECG and to the online gas analysis system (Quark CPET, Cosmed, Rome, Italy). Borg’s scale for rating of perceived exertion (RPE) [29] was taken immediately at the end of every session. Data were averaged at 5 s intervals and extracted to a commercially available program (Microsoft Excel, Office 365 Plus, Microsoft Corporation, Redmond, WA, USA) for further analysis. The variables calculated included mean values of oxygen uptake (VO_2_), carbon dioxide output (VCO_2_), respiratory exchange ratio (RER), minute ventilation (V_E_), ventilatory equivalent for carbon dioxide (V_E_/VCO_2_), heart rate (HR) and O_2_ pulse. Total oxygen uptake (VO_2_ sum), total ventilation (V_E_ sum), the total time spent > AT, >70%, >80% and >90% VO_2peak_ during exercise were also calculated.

### 2.5. Statistical Analysis

The data were analyzed with IBM SPSS statistics software version 26.0. Continuous variables are presented as means ± SD. Descriptive statistics (mean and standard deviation) were used for the baseline characteristics of the participants. The Shapiro–Wilk test was used to test the normality of distribution. A repeated measurement analysis of variance (ANOVA) with Sidak post-hoc comparisons was used to examine the differences between the three protocols. Partial η^2^, as a measure of effect size, was also reported. Power value was calculated with the statistics software. Due to the small sample size, non-parametric Friedman test with Wilcoxon tests (and appropriate adjustment of the *p* value) for post-hoc comparisons were also employed. Significance was set at the level of *p* < 0.05.

## 3. Results

Clinical and physiological characteristics of the patients are presented in Table 1. The cardiorespiratory responses from the CPX between the three protocols are exhibited in Table 2. Respiratory variables (V_E_ and relative VO_2_) were significantly lower at CON_50_ compared to CON_70_ and HIIT (*p* < 0.001). VO_2_ sum and V_E_ sum were significantly higher in CON_50_ compared to HIIT and CON_70_ and lower in CON_70_ compared to HIIT (*p* < 0.001). No differences were identified between the three protocols for VE/VCO_2_ (*p* > 0.05). Mean hemodynamic parameters (O_2_pulse, absolute and relative HR) were significant lower for CON_50_ compared to CON_70_ and HIIT (*p* < 0.001). No differences were found between CON_70_ and HIIT (*p* > 0.05).

No differences between the three protocols were found for the time spent > AT (*p* > 0.05; Table 3). Time spent > 70% VO_2peak_ was significantly higher to CON_70_ compared to HIIT (*p* < 0.01). Time spent > 80% and >90% VO_2peak_ was higher in HIIT compared to CON_50_ (*p* < 0.001). No differences found between HIIT and CON_70_ for the time spent > 80% (*p* > 0.05). For the time spent > 90%, a trend found between HIIT and CON_70_ (6.3 ± 5.0 min vs. 2.6 ± 4.9 min; *p* = 0.09). Mean intensity as a percentage of VO_2peak_ was lower on CON_50_ compared to CON_70_ and HIIT (*p* < 0.001; Table 2). In CON_70_, patients exercised at a higher mean percentage of VO_2peak_ compared to HIIT (*p* < 0.05).

The completion rate was 100% for the three protocols with no adverse events. The total exercise time was 69.8% higher to CON_50_ compared to CON_70_ (56.8 ± 4.3 min vs. 27.4 ± 0.3 min, *p* < 0.001) and 58.7% higher in CON_50_ to HIIT (56.8 ± 4.3 min vs. 31 min, *p* < 0.001). When HIIT was compared with CON_70_, there was a 12.3% difference with more time spent on HIIT (31 min vs. 27.4 ± 0.3 min, *p* < 0.001). No differences were identified between the three protocols in terms of total work performed (CON_50_: 22,011 ± 7757 J; CON_70_: 22,025 ± 8237 J; HIIT: 22,193 ± 7968 J; *p* = 0.61; partial η^2^: 0.369; observed power: 0.363), which confirms that the protocols successfully matched for the total work.

Significant between protocol differences were observed for the RPE (HIIT: 14.2 ± 1.8; CON_70_: 11.9 ± 2.9; CON_50_: 11.1 ± 2.9; *p* = 0.024; partial η^2^: 0.419; observed power: 0.411). HIIT tended to be higher than CON_50_ (*p* = 0.10); not any other differences were found between CON_50_ with CON_70_ (*p* = 0.70) and between HIIT with CON_70_ (*p* = 0.26).

When non-parametric analysis was performed, similar results for total and post-hoc comparisons were observed. In addition, the time spent > 90% VO_2peak_ was significantly higher in CON_70_ compared to CON_50_ (*p* < 0.01) (Table 3). Similarly, to previous instances the time spent > 90% VO_2peak_, tended to be higher in HIIT compared to CON_70_ (*p* < 0.022 for post-hoc comparison). HIIT tended to be somewhat lower than CON_70_ in terms of VO_2_ (mL∙kg^−1^∙min^−1^) (*p* = 0.028 for post-hoc comparison). CON_70_ tended to be higher than CON_50_ in terms of RER (*p* = 0.025 for post-hoc comparison). Finally, the Friedman test showed only some tendency (*p* = 0.16) for RPE.

## 4. Discussion

The aim of this study was to compare the acute cardiorespiratory responses between a HIIT, a high intensity and a low intensity-continuous protocol in CHF patients. The main findings were that HIIT and CON_70_ elicited higher cardiorespiratory responses compared to CON_50_ with significantly less exercise time. Moreover, with HIIT and CON_70,_ patients exercised longer at >80% and >90% of VO_2peak_. The completion rate too was 100% for the three protocols without any adverse events.

In CHF patients with left ventricular dysfunction, there are several central hemodynamic abnormalities during exercise, such as a reduction in cardiac output and an increase in end diastolic and end systolic volumes [30]. The central hemodynamic responses during exercise depend on the loading conditions, heart rate and the intrinsic ability of the heart to pump [31]. In the present study, the hemodynamic responses (HR, O_2_ pulse) were higher in HIIT and CON_70_ compared to CON_50_, with no significant differences between HIIT and CON_70_, which indicates similar cardiac stress between them. Moreover, based on O_2_ pulse, which reflects the responses of stroke volume and the arteriovenous oxygen difference, it can be supported that there was no difference between HIIT and CON_70_ in the ability of the heart to supply enough blood and the muscles to extract oxygen. Normandin et al. [21] showed that a HIIT protocol (30 s of cycling at 100% peak power output with 30 s passive recovery for 2 × 8 min) had similar mean HR and O_2_ pulse values with an iso-caloric MCT protocol in CHF patients. Tschakert et al. [22] found no differences in mean HR between a matched-load and time MCT, a short duration HIIT and a long duration HIIT protocol similar to ours, in CAD patients. Tomczak et al. [32] examined the acute responses in the post exercise biventricular function using the Norwegian HIIT protocol [9] in non-ischemic CHF patients. They found that left ventricular end systolic volume decreased by 6% and LVEF was increased by 2.4%, 30 min after the end of the exercise session because of the maintenance of contractility and the reduction of LV afterload.

CHF patients are characterized by exertional dyspnea accompanied by abnormal increased ventilation [33,34]. When examining the VO_2_sum and V_E_sum, differences were identified between the three protocols with highest values observed in CON_50_. This can be explained by the differences in total time with mean time in CON_50_ being much higher. In terms of mean pulmonary responses, HIIT and CON_70_ elicited significantly higher values compared to CON_50_ without any differences between them. In a previous study, mean ventilation was lower compared to our data, with no differences between an MCT and HIIT, as well no differences in RPE but a higher completion rate for HIIT [21]. In another study [18], it was found that in the MCT protocol (70% peak power output), mean ventilation was higher than a HIIT (2 × 10 min of 15 s cycle at 100% peak power output interspersed by 15 s passive recovery); the authors reported that the patients preferred the HIIT rather than MCT because of the less sensation of breathlessness [18]. The potential mechanisms for the increased ventilatory drive are an increase in physiological dead space (V_D_/V_T_) and an earlier onset of lactic acidosis [35]. Changes observed in V_D_/V_T_ have been attributed to ventilation perfusion mismatching and an abnormal breathing pattern (lower tidal volume, high breathing frequency) [35]. Moreover, V_E_/VCO_2,_ which is a marker of breathing efficiency during exercise [34], was also similar between the three protocols. To support this notion, neither of the three protocols exceeded an RER > 1, indicating no hyperventilation or excess of CO_2_ in either protocol.

In the present study, it was demonstrated that the patients in HIIT and CON_70_ exercised at a higher %VO_2peak_ compared to CON_50_, with patients in CON_70_ protocol exercising at a higher %VO_2peak_ intensity compared to HIIT. This is in line with previous studies that despite the relatively low intensity of continuous exercise, the patients had higher metabolic demand as reflected by the higher relative and/or absolute VO_2_ values [18,20,21]. A reason why these protocols had higher %VO_2peak_ in comparison to the intensity originally prescribed may be due to the slow component of VO_2_. The patients exercised an appreciable time above their ventilatory threshold; therefore, the exercise intensity might be considered as heavy. Above the heavy domain, the slow component drives the VO_2_ above that predicted for the work rate [36]. That could be also the case for the CON_50_ protocol, in which the patients also exercised above ventilatory threshold for some time. Moreover, in both HIIT and CON_70_, more time was spent at >80% and >90% VO_2peak_, suggesting that both HIIT and CON_70_ protocols produced high physiological stimuli. This, in turn could potentially lead to higher adaptations than CON_50_ in the long-term. Based on post-hoc comparisons, time spent > 90% VO_2peak_ tended to be somewhat higher (3.7 min) in HIIT compared to CON_70_. Although the lack of significance may be possibly related to the small sample size, this finding may be of clinical importance because previous studies have highlighted the importance of the time spent in higher intensities for optimal cardiovascular improvements. Kemi et al. [37] evaluated the effects of different exercise intensities in VO_2max_, cardiomyocyte contractility and arterial function in rats. They found that the group that exercised in the higher intensities elicited larger improvements in VO_2max,_ which was correlated with an increase in cardiomyocyte size and function; both protocols improved endothelium dependent carotid dilations. Moholdt et al. [5] categorized 112 coronary artery disease patients based on the exercise intensity after a 12-week program. They observed that the patients who exercised > 92% HR_peak_ elicited the highest improvements in VO_2peak_ and concluded that, although all patients exercised with a high intensity protocol, the higher exercise intensity was an important component to improve VO_2peak_.

All the sessions were completed successfully without any adverse events. Therefore, all three protocols employed in this study were safe. RPE was not found to be different between protocols; beyond tendencies, the exact differences were not able to be defined, likely due to the small sample size. In any case, RPE was within acceptable limits. When looking the HIIT and CON_70_, the lack of difference between them could also be attributed to the fact that the protocols had the same cardiopulmonary responses without overloading the ventilatory system, in line with previous data [17,21]. However, in the present study CON_70_ and CON_50_ protocols had lower RPE compared to previous research [21]. In HIIT, RPE was slightly lower compared to previous studies [17,21]. This can be explained by differences in total exercise time, passive recovery and shorter exercise intervals.

This pilot study provides important clinical information regarding exercise prescription in CHF patients and could at least partly explain the lack of differences between a HIIT and an MCT protocol in CHF and CAD patients, respectively [13,14]. An important limitation of SMARTEX-HF was that a large percentage of the cardiac patients did not exercise in their prescribed intensity; HIIT patients exercised below their prescribed intensity and MCT patients exercised above. In SAINTEX-CAD [13], the mean intensity the patients exercised in the MCT protocol was 80% HR_peak_, which could not be considered moderate. Given also that none of the MCT patients terminated the exercise prematurely indicates that they were able to sustain such a high intensity continuously [13]. This comes in agreement with the present data where CHF patients safely sustained a higher intensity continuous regime more time efficiently compared to a matched work lower intensity continuous protocol and yielded similar cardiorespiratory responses as the high volume HIIT.

In an extended exercise prescription perspective, both HIIT and CON_70_ could be used (interchangeably) in exercise rehabilitation programs to induce optimal benefits. From a behavioral point of view, both demanded appreciably shorter time to accomplish than CON_50_; this will assist to potentially improve exercise compliance and satisfaction given the benefit of shorter exercise time but with no significant difference in RPE. From a physiological point of view overall, they similarly induced a higher acute cardiorespiratory stimulus than CON_50_, an exercise regime frequently employed in cardiac rehabilitation [38], potentially resulting in higher long-term cardiorespiratory gains [5,8]. CON_70_ in fact resulted in a somewhat higher average VO_2peak_ percentage value than HIIT, while HIIT may result in somewhat higher accumulated exercise time at close-to-maximum intensities. Whether any of these HIIT and CON_70_ protocols turn out to be more advantageous in the long-term remains to be decided. In any case and from a clinical standpoint, these differences sound as rather minor and of a doubtful practical importance. In addition, the data presented suggest intensity to be an essential exercise characteristic for continuous regimes.

The present study had some limitations. It included a small number of patients, all men, with predominant functional class NYHA I-II. Therefore, the results cannot be generalized for all the CHF patients, especially those of lower functional capacity (NYHA III/IV). However, it was a pilot study with proper power for most variables examined to provide some insight into the cardiorespiratory responses of different aerobic regimes, extensively used in recent years.

## 5. Conclusions

In the present pilot study, both a high volume HIIT and a high intensity continuous protocol (CON_70_) elicited a strong physiological stimulus, without any ventilatory limitations compared to lower intensity continuous exercise protocol (CON_50_) matched for total work. A high intensity continuous regime is safe and tolerable and more time efficient compared to a low intensity continuous regime.

## Figures and Tables

**Table 1:** Anthropometric, clinical and cardiopulmonary exercise testing characteristics.

(*n* = 10, LVEF < 45%)	Values ^a^
Age (Years)	55.1 ± 16.2
Men	10 (100%)
Weight (kg)	84.9 ± 21.4
Body mass index (kg/m^2^)	27.9 ± 4.7
Height (cm)	172.9 ± 9.4
VO_2peak_ (mL∙kg^−1^∙min^−1^)	19.8 ± 4.5
VO_2peak_ (% VO_2peak_ predicted)	65.7 ± 10.1
AT (mL∙kg^−1^∙min^−1^)	12.3 ± 3.2
AT (% VO_2peak_)	62 ± 7
HR_peak_ (beats·min^−1^)	122 ± 14
NYHA classification I/II/III, *n* (%)	2 (20%)/7 (70%)/1 (10%)
LVEF	33.8 ± 5.3
Etiology of heart failure	
Dilated cardiomyopathy	3 (30%)
Ischemic heart disease	6 (60%)
Valvular	1 (10%)
Medications	
ACE inhibitors	8 (80%)
β-blockers	8 (80%)
Loop diuretics	10 (100%)
Antiplatelets/Anticoagulants	6 (60%)/1 (10%)
Spironolactone	4 (40%)
Nitrates	2 (20%)
Amiodarone	2 (20%)
ICD device	2 (20%)

Abbreviations: ACE, angiotensin converting enzyme; ARBs, angiotensin receptor blockers; AT, anaerobic threshold; HR_peak_, heart rate peak; ICD, implantable cardioverter defibrillator; LVEF, left ventricular ejection fraction; NYHA, New York Heart Association; VO_2peak_, peak oxygen consumption. ^a^ Data presented as mean ± SD or *n* (%).

**Table 2:** Cardiorespiratory responses during HIIT, CON70 and CON50.

	HIIT	CON_70_	CON_50_	*p* Value	Partial η^2^	Observed Power
VO_2_ sum (L)	38.7 ± 11.1	36.2 ± 10.4 ^a^	53.5 ± 10.2 ^a,b^	<0.001	0.915	1.000
V_E_ sum (L)	1274.1 ± 280.6	1155.3 ± 263 ^a^	1574 ± 241.5 ^a,b^	<0.001	0.795	1.000
V_E_ (L∙min^−1^)	41.1 ± 2.8	42.0 ± 2.8	27.8 ± 1.5 ^a,b^	<0.001	0.901	1.000
VO_2_ (mL∙kg^−1^∙min^−1^)	14.9 ± 3.3	15.7 ± 3.1	11.5 ± 2.1 ^a,b^	<0.001	0.899	1.000
VO_2_ (% VO_2peak_)	75.9 ± 6.4	79.8 ± 5.9 ^a^	58.8 ± 4.9 ^a,b^	<0.001	0.960	1.000
VE/VCO_2_	34.8 ± 3.9	34.5 ± 4.4	34.1 ± 4.2	0.755	0.031	0.084
RER	0.95 ± 0.04	0.94 ± 0.06	0.87± 0.04 ^a^	0.002	0.486	0.934
O_2_ pulse (mL∙beat^−1^)	13.6 ± 3.1	14.0 ± 2.5	11.9 ± 2.2 ^a,b^	0.002	0.599	0.948
Heart rate (beats∙min^−1^)	91.3 ± 12.6	93.4 ± 13.9	80.6 ± 11.7 ^a,b^	<0.001	0.716	1.000
Heart rate peak (%)	74.7 ± 8.8	76.8 ± 11.4	67.3 ± 9.2 ^a,b^	0.001	0.518	0.959

Abbreviations: O_2_ pulse; oxygen pulse, RER; respiratory exchange ratio, VE; minute ventilation, VE sum, total ventilation, VE/VCO_2_; ventilatory equivalent for carbon dioxide; VO_2_ sum; total oxygen uptake; VO_2_; oxygen uptake. Data reported as mean ± SD or *n* (%). ^a^ Significant compared to HIIT (*p* < 0.05). ^b^ Significant compared to CON70 (*p* < 0.01).

**Table 3:** Time (min) spent > AT, >70%, >80% and >90% VO_2peak_.

	HIIT	CON_70_	CON_50_	*p* Value	partial η^2^	Observed Power
>AT	23.8 ± 6.1	25.0 ± 3.7	24.2 ± 21.2	0.962	0.040	0.055
>70% VO_2peak_	19.3 ± 3.8	24.6 ± 2.9 ^a^	3.1 ± 5.4 ^a,b^	<0.001	0.930	1.000
>80% VO_2peak_	13.7 ± 5.0	15.5 ± 10.3	0.2 ± 0.7 ^a,b^	<0.001	0.723	1.000
>90% VO_2peak_	6.3 ± 5.0	2.6 ± 4.9 ^a^	0.02 ± 0.0 ^a,b^	0.03	0.482	0.930

Abbreviations: AT; anaerobic threshold, HIIT; high intensity interval training, VO_2peak_; peak oxygen uptake. Data reported as mean ± SD. ^a^ Significant compared to HIIT (*p* < 0.01). ^b^ Significant compared to CON_70_ (*p* < 0.01).

## Data Availability

Available from corresponding author upon reasonable request.
